# Engineering tandem CD33xCD146 CAR CIK (cytokine-induced killer) cells to target the acute myeloid leukemia niche

**DOI:** 10.3389/fimmu.2023.1192333

**Published:** 2023-05-25

**Authors:** Gaia Alberti, Corinne Arsuffi, Alice Pievani, Domenico Salerno, Francesco Mantegazza, Francesco Dazzi, Andrea Biondi, Sarah Tettamanti, Marta Serafini

**Affiliations:** ^1^ Tettamanti Center, Fondazione IRCCS San Gerardo dei Tintori, Monza, Italy; ^2^ School of Medicine and Surgery, BioNanoMedicine Center NANOMIB, Universita di Milano-Bicocca, Vedano al Lambro (MB), Italy; ^3^ School of Cardiovascular Sciences, King’s College London, London, United Kingdom; ^4^ Dipartimento di Medicina e Chirurgia, Università degli Studi Milano-Bicocca, Monza (MB), Italy; ^5^ Pediatrics, Fondazione IRCCS San Gerardo dei Tintori, Monza, Italy

**Keywords:** acute myeloid leukemia, tandem CAR, cytokine-induced killer (CIK cells), AML niche, MSCs (mesenchymal stem cells)

## Abstract

In acute myeloid leukemia (AML), malignant stem cells hijack the normal bone marrow niche where they are largely protected from the current therapeutic approaches. Thus, eradicating these progenitors is the ultimate challenge in the treatment of this disease. Specifically, the development of chimeric antigen receptors (CARs) against distinct mesenchymal stromal cell subpopulations involved in the maintenance of leukemic stem cells within the malignant bone marrow microenvironment could represent a new strategy to improve CAR T-cell therapy efficacy, which is still unsuccessful in AML. As a proof of concept, we generated a novel prototype of Tandem CAR, with one specificity directed against the leukemic cell marker CD33 and the other against the mesenchymal stromal cell marker CD146, demonstrating its capability of simultaneously targeting two different cell types in a 2D co-culture system. Interestingly, we could also observe an *in vitro* inhibition of CAR T cell functionality mediated by stromal cells, particularly in later effector functions, such as reduction of interferon-gamma and interleukin-2 release and impaired proliferation of the CAR^+^ effector Cytokine-Induced Killer (CIK) cells. Taken together, these data demonstrate the feasibility of a dual targeting model against two molecules, which are expressed on two different target cells, but also highlight the immunomodulatory effect on CAR CIK cells exerted by stromal cells, confirming that the niche could be an obstacle to the efficacy of CAR T cells. This aspect should be considered in the development of novel CAR T cell approaches directed against the AML bone marrow niche.

## Introduction

The recent clinical success of CAR (Chimeric antigen receptor) T cell immunotherapy in the context of B-cell malignancies has opened a new route of investigation also towards AML. The development of CAR T cell therapy in the context of AML is still in its infancy due to heterogeneity of the disease, the lack of a suitable target antigen and the leukemia protective role of the tumor microenvironment (TME). Indeed, there are many evidences that AML patients have an altered TME, where malignant cells establish specific interdependencies with the surrounding niche components by engaging a bidirectional crosstalk and modulating important mechanisms leading to AML proliferation, survival, resistance to chemotherapy, and immune evasion (1, 2). Studies on the biological features of the altered TME have demonstrated its critical contribution to disease formation and progression, thus making TME a promising target to improve AML treatment (3). AML blasts can actively shape their TME that become supportive for malignant cells and detrimental for normal hematopoiesis (4–6). Among the non-hematopoietic cell components of the niche, mesenchymal stromal cells (MSCs) recover a crucial role in sustaining leukemogenesis (7). Altered MSCs can support leukemic cells through several mechanisms, including releasing of pro-survival factors, ligand-receptor interactions, exchanges of organelles, and modifications of leukemic metabolism (8). Taken together, these modifications contribute to establish a shelter for the therapy-resistant leukemic stem cells (LSCs) and AML blasts. Notably, increasing evidence of the TME adverse impact in the efficacy of CAR T cell immunotherapy are emerging both in clinical settings of solid (9, 10) and hematological (11) malignancies. Among all the factors that could influence the permanence and activity of CAR T cells in the bone marrow (BM), MSCs were shown to play an immunosuppressive effect on cells of both innate and adaptive immunity. MSCs are indeed capable of inhibiting B cell functions and differentiation (12), preventing monocyte differentiation (13) and altering the cytokines milieu in favor of an anti-inflammatory profile (14–16). Moreover, MSCs were found to inhibit T cell proliferation (17, 18) and IL-2 and TNFα production (19), inducing T regulatory cells (Tregs) to exploit their inhibitory functions (20, 21). Immunosuppression by MSCs is mediated also by paracrine mechanisms, including indoleamine 2,3-dioxygenase (IDO), nitric oxide, transforming growth factor β1, prostaglandin E2, and IL-10 release (22, 23). Due to all these reasons, we aimed to develop a prototype of bispecific CAR that would be able to simultaneously recognize both the AML blasts and the MSCs within the AML niche. Preclinical studies in solid tumors have shown promising results in the targeting of stromal components with CAR T cells (24, 25). Among the possible targets, Melanoma Cell Adhesion Molecule (MCAM)/CD146 represents a useful tool since it has been found expressed by a subpopulation of BM human stromal cells that have a pivotal role in the establishment of the HSC niche (26) and are implicated in resettlement, survival and growth of tumor cells in human BM niches recreated *in vivo* (27–29). Specifically, in the AML context, Kumar and colleagues described an increase in the frequency of CD146^+^ cells in the stromal compartment of leukemia-engrafted mice BM (6). Furthermore, CD146 on stromal cells has been recently involved in promoting malignant myeloid cell proliferation and survival (30). Regarding the targeting of AML blasts, CD33 molecule is one of the most validated immunotherapeutic targets in clinical studies (31), being overexpressed in the majority of AML blasts (32) and on LSCs (31).

In this work, we first developed a bispecific Tandem CAR, cloning an anti-CD146 scFv together with an anti-CD33 scFv, to address the hypothesis of targeting both AML blasts and MSCs within the BM niche. Tandem CD33/CD146.CAR CIK cells displayed a specific *in vitro* anti-leukemic efficacy against CD33 positive cell line and, concomitantly, a cytotoxic effect against a CD146 positive cell line maintained in co-culture conditions with leukemic cells. Moreover, when tested against primary MSCs and stromal cell lines we highlighted a potent immunosuppression by means of decreased proliferation and cytokine production of CAR CIK cells.

## Materials and methods

### Plasmids

Humanized mouse anti-human CD146 clone OI-3 (kindly provided by Oncoinvent AS) was used to generate six CD146.CAR molecules. Each different design was optimized with three different spacer (CH2mutCH3, CH3, CD8stalk) and two opposite VH (heavy chain variable domain) and VL (light chain variable domain) orientations. The high-affinity, humanized rat anti-human CD33 single chain fragment variable-Fv-(L-(gly4ser)4-H was generated using UCB’s Selected Lymphocyte Antibody Method (kindly provided by Dr. Helene Finney, UCB Celltech, Slough, UK). Two different Tandem CARs were generated (CD33/CD146.CAR and CD146/CD33.CAR) using scFvs from CD33.CAR and the optimal CD146.CAR. CD146.CAR, CD33.CAR and Tandem CAR molecules were codon optimized and cloned in frame with CD28transmembrane-CD28-OX40-CD3z, CH3CH3-CD28transmembrane-CD28-OX40-CD3z and CH2mutCH3-CD28transmembrane-41BB-CD3z respectively, in the Sleeping Beauty (SB) pT4 vector, together with SB100X transposase (kindly provided by Dr. Zsuzsanna Izsvak).

### Manufacturing

Cytokine-induced killer (CIK) cells were generated from healthy donor peripheral blood mononuclear cells (PBMCs) according to our protocol (33) by adding IFN-γ (1,000U/ml, Dompè Biotec) and subsequently stimulating with IL-2 (300U/ml, Chiron BV) and OKT-3 (50ng/ml Janssen-Cilag). After 24h, PBMCs were nucleofected with SB transposon and transposase DNA plasmids according to the manufacturer’s instructions using 4D-Nucleofector TM device (Lonza). At the end of the differentiation protocol, mature CIKs typically are almost entirely CD3^+^ T lymphocytes expressing CD8 and CD56 and display an effector memory phenotype.

### Cell lines

TIME cell line was provided from American Type Culture Collection (ATCC). Cells were cultured with Vascular Cell Basal Medium (ATCC^®^PCS-100-030), supplemented with Microvascular Endothelial Cell Growth Kit-VEGF (ATCC^®^PCS-110-041), which contains several purified human recombinant (rh) growth factors and combined with 10 mM L-glutamine, 0.75 U/mL of heparin sulfate, 1 mg/mL of hydrocortisone hemisuccinate, 5% FBS, and 50 mg/mL of ascorbic acid (ATCC). Medium renewal was done every 3 days and cells were split at 80-90% confluence using Trypsin-EDTA for Primary Cells (ATCC PCS-999-003). Cells were cultured at 37°C in 5% CO2. TIME cells are 100% CD146 positive. KG-1 and HS-27A cell lines were provided from ATCC and HS-5 cell line was kindly provided by Dr. Monica Casucci (HSR, Milan). Cell lines were cultured in RPMI-1640 (EuroClone) with 1% of L-glutamine (Invitrogen) and 1% penicillin-streptomycin (Invitrogen), 10% FBS (Gibco) at 37°C in 5% CO2. KG-1 cell density was maintained at 0,3x10^6^ cells/ml splitting twice a week. KG-1 cells are 100% CD33 positive. HS-5 and HS-27A medium renewal was done twice a week and cells were split at 80-90% confluence using trypsin-EDTA (Invitrogen).

### Mesenchymal stromal cells

Mononuclear cells (MNCs) from BM aspirates of pediatric AML patients at diagnosis and of healthy donors (HDs) were isolated using a Ficoll-Paque TM Plus (GE Healthcare) density gradient separation. To obtain MSCs, MNCs were seeded at a density of 2x10^5^ cells/cm^2^ in complete culture medium: DMEM (Dulbecco Modified Eagle Medium) low glucose (1 g/L, Invitrogen), supplemented with 1% of L-glutamine and 1% penicillin-streptomycin, 10% FBS at 37°C in 5% CO^2^. Non-adherent cells were removed 48 hours after initial plating by washing with PBS and the complete culture medium was changed every 3 or 4 days. At 70% of confluence, MSCs were harvested using trypsin-EDTA and for subsequent expansion cells were re-plated at 2x10^3^ cells/cm^2^.

MSC cultures were characterized by assessing morphology, immunophenotype, and tri-lineage differentiation potential (adipogenic, osteogenic and chondrogenic differentiation), and used for our experiments until passage 8.

MSCs were obtained from BM collected from healthy donors and pediatric AML patients. The studies involving human participants were reviewed and approved by the Institutional Review Board of the Ethical Committee of San Gerardo Hospital. Written informed consent to participate in this study was provided by the patients or their guardians.

### Flow cytometry

CIK cells were tested for the expression of CD3 (clone SK7), CD8 (clone RPA-T8), CD4 (clone SK3), CD56 (clone B159), CD62L (clone DREG-56) and CD45R0 (clone UCHL1) using specific antibodies (BD Bioscience). CD146.CAR expression was detected using a recombinant human MCAM/CD146 protein with a Fc (BioTechne), before proceeding with secondary staining with an anti-human IgG Fc Alexa Fluor 647-conjugated mAb (LiStarFish). CD33.CAR expression was detected using a recombinant human sialic acid binding Ig-Like Lectin 3/Siglec-3/CD33 protein with a Fc and a 6xHis tag at the C-terminus (C-Fc-6His) (Gentaur), before proceeding with secondary staining with a 6xHis Tag mAb (AD1.1.10) (Invitrogen) FITC-conjugated. Tandem CAR molecules were stained with both chimera proteins and with both secondary antibodies. Target cells were assessed for CD146 (clone P1H12) and CD33 (clone HIM3-4) expression. Anti-human IFNγ (clone B27), IL-2 (clone MQ1-17H129) and Ki-67 (clone B56) mAbs (BD) have been used to assess CIK cells cytokine production and proliferation. Cell death was detected using the GFP-Certified™ Apoptosis/Necrosis detection kit (Enzo Life Sciences), according to the manufacturers’ instructions. Target cells were also labeled with FITC- and PE- Cell Tracker (Invitrogen) or CellTrace™ Violet (Thermofisher Scientific). Samples were acquired using the FACSCanto II flow cytometer (BD) and data were analyzed using BD FACS DIVA software version 6.1.3.

### CAR^+^ cells sorting assay

CAR CIK positive cells purification was performed by magnetic separation on LS MACS^®^ column placed in the magnetic field of a MACS Separator, using anti-APC MicroBeads (MiltenyiBiotec) added after staining for CD33.CAR.

### Short term cytotoxicity assay

To evaluate the killing ability of both unmodified and CAR-redirected CIK cells, they were co-cultured for 4 hours with target cells (previously labeled with FITC-Cell Tracker or Violet CellTrace) at an effector-target (E:T) ratio of 5:1. At the end of the incubation, target cells killing was measured by flow cytometry, after annexin V and Necrosis Detection Reagent (NDR) staining. The percentage of killed cells was determined adding the percentage of PE^+^/Annexin V^+^/NDR^−^ cells to that of PE^+^/Annexin V^+^/NDR^+^ cells in co-culture with the effectors compared to target cells alone.

### Proliferation assay

The proliferation ability of CD146.CAR, CD33.CAR and CD33/CD146.CARCIK cells was evaluated after co-culture with target cells (E:T ratio of 1:1). After 72 hours of co-culture, cells were harvested and stained for CD3 and CAR. Hence, intracellular staining for Ki-67 was performed using the BD Cytofix/Cytoperm kit. Specimens were then analyzed by flow cytometry.

### Long-term proliferation assay

Unmodified and CAR^+^ CIK cells proliferation ability was also evaluated in a long-term assay, after 7 days of co-culture with KG-1 cells (previously labeled with CellTrace Violet or PE-cell Tracker) (E:T ratio of 1:10), in the presence or not of an MSCs layer. After the incubation, a staining with CD3 antibody was performed to identify CIK cells population and CIK cells proliferation ability was evaluated by flow cytometry quantitative analysis. The fold increase after engagement with KG-1 cells was calculated using the following formula:


$$\frac{Number\;of\;CIK\;cells\;after\;the\;co-culture\;with\;KGI\;cells,\;normalized\;on\;beads\;number}{Number\;of\;CIK\;cells\;after\;the\;culture\;alone,\;normalized\;on\;beads\;number}$$

### Intracellular cytokine production assay

CAR-redirected CIK cells ability to produced cytokines was evaluated following a stimulation with the different target cells at (E:T ratio of 1:3). After 2 hours and 30 minutes of incubation, BD GolgiStop™ was added, and the co-culture was then maintained for an additional period of 2 hours and 30. Then cells were collected and stained for CD3 and CAR surface molecules. Hence, intracellular cytokine staining for IL-2 and IFN-γ was performed using the BD Cytofix/Cytoperm kit. Specimens were then analyzed by flow cytometry.

### Operetta CLS image acquisition

Time-dependent apoptosis mediated by VL-VH Long CD146.CAR CIK cells against TIME, MSCs and HS-27A was evaluated using Operetta CLS (PerkinElmer, Waltham, MA, USA), a high throughput spinning disk confocal microscope equipped with eight emission LED sources ranging from the near ultraviolet 360nm to the far red 650nm working at controlled temperature and CO2 concentration. The confocal images acquisition was performed using the 40X water immersion objective (numerical aperture 1.1). Target cells were labeled with CellTracker™ Deep Red Dye (Thermofisher Scientific), CAR CIK cells were stained with CellTrace™ Violet (Thermofisher Scientific) and the cell death marker NucView^®^ 488 Green Caspase-3 (Biotium) was used to measure the cytotoxic activity of CAR CIK cells against CD146^+^ target cells. CIK cells were co-cultured for 8 hours with the target cells at an E:T ratio of 5:1 and images were taken every 5 minutes for the first 2 hours and for the subsequent 6 hours every 30 minutes. A custom-made MatLab (Mathworks inc. USA) based software provided the image analysis to extract the populated area of the different dyes.

### Statistical analysis

Data were analyzed using GraphPad Prism 9 software (GraphPad). Two-way ANOVA analysis with Benjamini and Hochberg’s multiple comparison, Two-way ANOVA analysis with Benjamini and Hochberg’s multiple comparison and Wilcoxon T-test were performed to determine the statistical significance of data. Reported values of the statistical analyses are the result of the evaluation of mean ± SD

## Results

### Engineering CD146.CAR CIK cells with VL-VH long isoform

**Figure 1:**
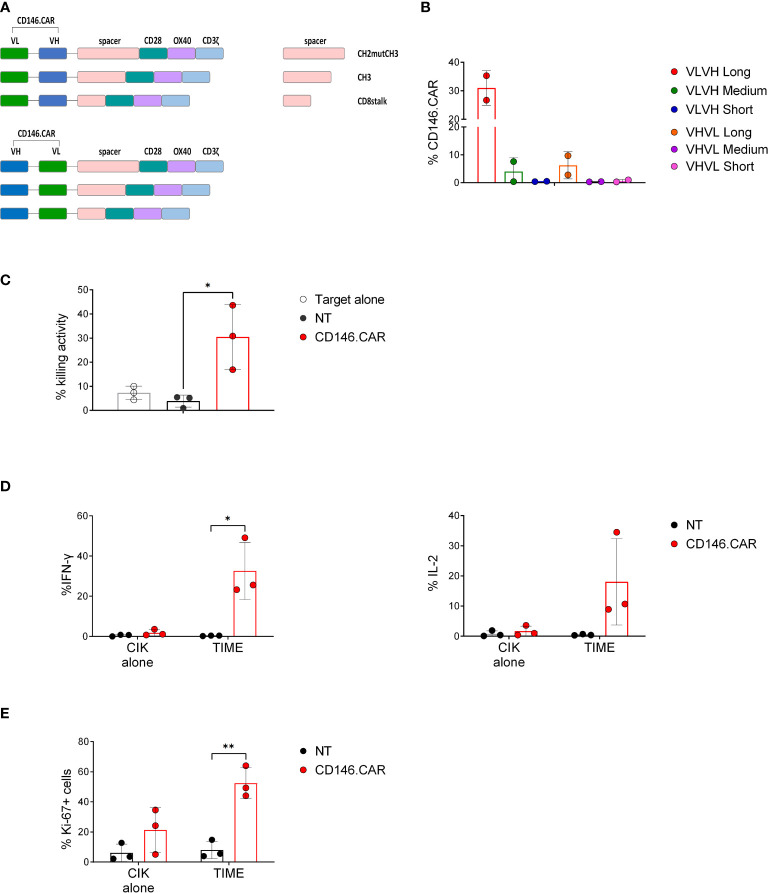
Selection and *in vitro* characterization of functional CD146.CARCIK cells. **(A)** Schematic diagram of CD146.CAR structure, including VL and VH domains of single chain fragment variable (scFv), the spacer sequences: CH2mutCH3 (Long), CH3 (Medium) and CD8stalk (Short), the CD28-OX40 co-stimulatory domains and CD3ζ signaling domain. **(B)** Percentage of expression of CD146.CAR molecules by CIK cells at day21 of culture, determined by FACS analysis (n=2). **(C)** Short-term cytotoxicity of untransduced (NT) and CD146.CAR CIK cells co-cultured with CD146^+^ adherent cell line (TIME) at effector:target (E:T) 5:1 ratio (n=3). **(D)** Cytokines production (IFN-γ and IL-2) by CD146.CAR CIK cells when engaged with TIME cells. IFN-γ and IL-2 were detected by intracellular staining after 5-hours co-culture at 1:3 E:T ratio(n=3). **(E)** Proliferation determined by intracellular Ki-67 staining of NT and CD146.CAR CIK cells after co-culture with TIME cells for 72 hours at 1:1 E:T ratio (n=3). (*P<0.05, **P<0.01, Two-way ANOVA analysis with Benjamini and Hochberg’s multiple comparisons test).

To generate a functional CD146.CAR, we firstly designed six third-generation CD146.CAR molecules that differ in the single chain fragment variable (scFv) orientation (VL-VH and VH-VL, conventional and unconventional respectively) and in the spacer sequences (CH2mutCH3, CH3, CD8stalk, named Long, Medium, Short). All the CD146.CAR molecules held the same co-stimulatory domains (CD28-OX40) and CD3ζ as signaling domain (**Figure 1A**).

Among all isoforms, VL-VH Long displayed the major expression of CD146.CAR (approximately 30%) on CIK cells at the end of the culture (**Figure 1B**). Therefore, this variant was selected for the subsequent *in vitro* functional analyses (namely CD146.CAR).

Indeed, CD146.CAR CIK cells displayed a high cytotoxicity against the CD146^+^ adherent cell line TIME, as compared to the non-transduced (NT) CIK cells (**Figure 1C**). Moreover, a higher production of interferon-gamma (IFN-γ) and interleukin-2 (IL-2), as well as a significant increase of Ki-67 expression (**Figure 1E**) were observed as compared to NT CIK cells, when CD146.CAR CIK cells were engaged with TIME cells (**Figure 1D**), demonstrating a specific target-dependent activation and proliferation.

### Tandem CAR molecule specific for CD33^+^ and CD146^+^ target cells

After having validated the functionality of CD146.CAR against TIME cell line, a Tandem construct was conceived to probe the concomitant dual targeting of both AML blasts and CD146^+^ stromal cells.

**Figure 2:**
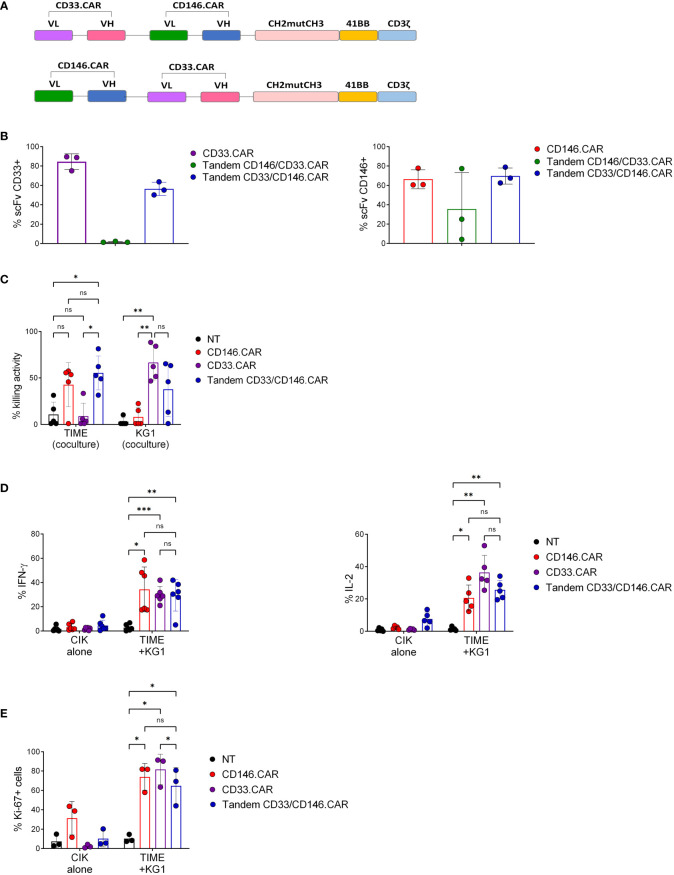
Developing second-generation Tandem CD33/CD146.CAR CIK cells able to recognize simultaneously CD33^+^ KG-1 and CD146^+^ TIME cells, and to display full CAR *in vitro* effector functions. **(A)** Schematic diagram of Tandem CAR molecules’ structure, including CD33 and CD146 scFvs in the different position, CH2mutCH3 (Long) as spacer sequence, 4-1BB as co-stimulatory domain and CD3ζ signaling domain. **(B)** Percentage of expression of CD33 and CD146 scFv by CIK cells at day 21 of culture, determined by FACS analysis(n=3). **(C)** Short-term cytotoxicity of untransduced (NT), single CD33- and CD146- CAR CIK cells and Tandem CD33/CD146.CAR CIK cells co-cultured with CD146^+^TIME and CD33^+^ KG-1 cells at 5:1 E:T ratio (n=3). **(D)** Cytokines production (IFN-γ and IL-2) by NT, single CD33- and CD146- CAR CIK cells and Tandem CD33/CD146.CAR CIK cells when engaged with TIME and KG-1 cells. IFN-γ and IL-2 were detected by intracellular staining after 5-hours co-culture at 1:3 E:T ratio (n=3). **(E)** Proliferation determined by intracellular Ki-67 staining of NT, single CD33- and CD146- CAR CIK cells and Tandem CD33/CD146.CAR CIK cells after co-culture with TIME cells and KG-1 for 72 hours at 1:1 E:T ratio (n=3). (*P<0.05, **P<0.01, ***P<0.001, Two-way ANOVA analysis with Benjamini and Hochberg’s multiple comparisons test). NS, not significant.

Therefore, we developed two second-generation Tandem CARs bearing CH3mutCH3 as spacer sequence, 4-1BB as co-stimulatory domain and CD3ζ as signaling domain. The molecules differ in the positioning order of CD33 and CD146 scFv, obtained from CD33.CAR and CD146.CAR, respectively (**Figure 2A**). Only Tandem CD33/CD146 CAR CIK cells reached optimal expression levels of both CD33 and CD146 scFv at the end of the culture (**Figure 2B**). This variant was further *in vitro* characterized by co-culture with both CD146^+^ TIME cells and CD33^+^ AML cell line (KG-1). Tandem CD33/CD146 CAR CIK cells showed a relevant killing activity against both target cells, comparable to CD33.CAR against KG-1 and CD146.CAR against TIME (**Figure 2C**). In addition, Tandem CD33/CD146.CAR CIK cells activation and proliferation were confirmed by enhanced IFN-γ and IL-2 (**Figure 2D**) and Ki-67 (**Figure 2E**) production. Taken together, these results demonstrated that Tandem CD33/CD146.CAR CIK cells were able to specifically recognize and be functional *in vitro* against both target cells (KG-1 and TIME).

### Immunosuppressive action of the niche stromal compartment on CAR CIK cells later effector functions

**Figure 3:**
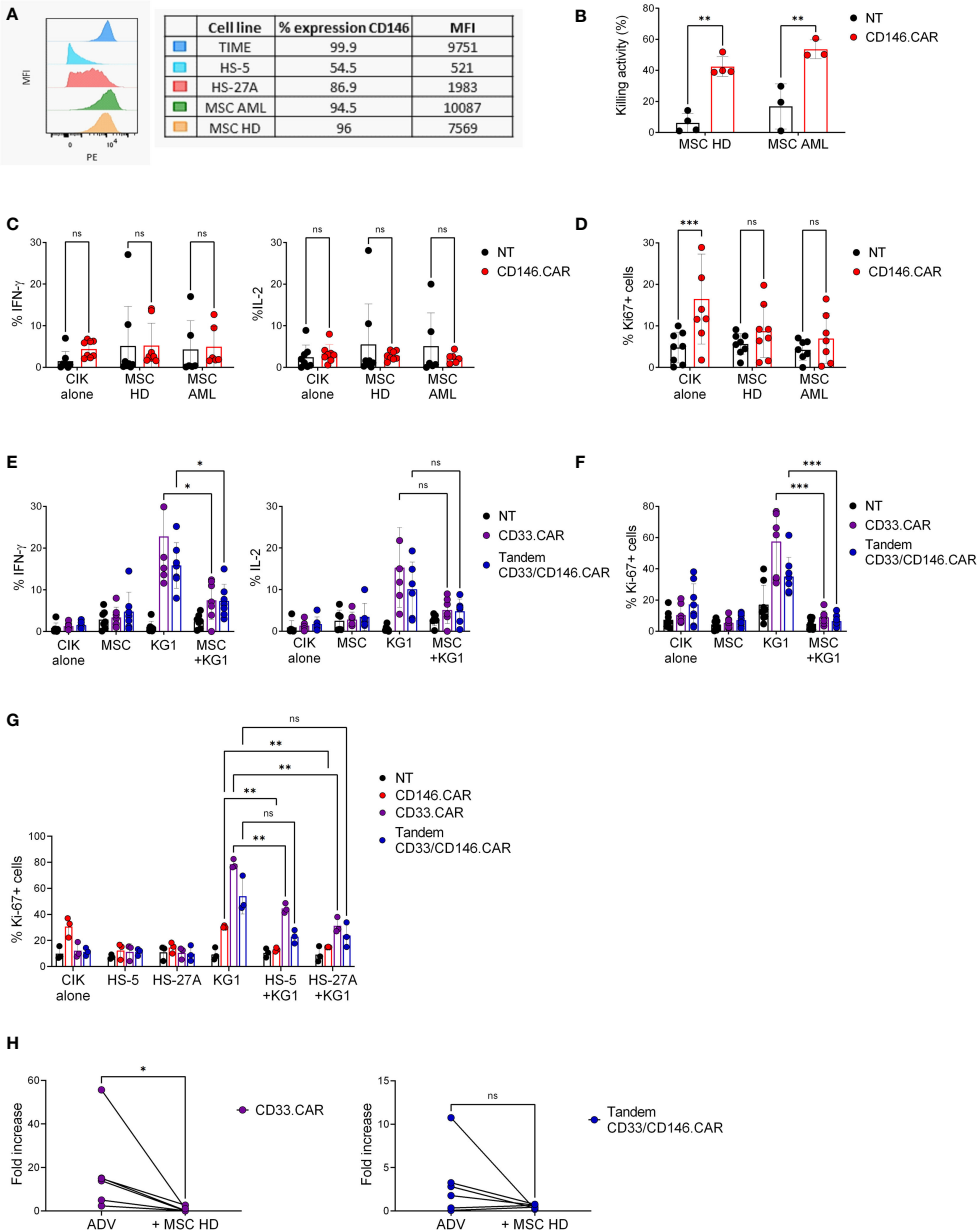
CD146^+^ MSCs and stromal cell lines display long-term immunosuppressive action against CAR CIK cells. **(A)** Expression levels of CD146 on target cells (TIME, HS-5, HS-27A, pediatric MSC-AML and MSC-HD), analyzed as percentage of positivity and MFI by flow cytometric analysis. **(B)** Short-term cytotoxicity of untransduced (NT) and CD146.CAR CIK cells co-cultured with CD146^+^ HD- and AML-MSCs (E:T ratio 5:1) (HD-MSC n=4, AML-MSC n=3). **(C)** Cytokines production (IFN-γ and IL-2) by CD146.CAR CIK cells when engaged with CD146^+^ HD- and AML-MSCs (E:T ratio1:3) (n=8). **(D)** Proliferation of NT and CD146.CAR CIK cells after co-culture with CD146^+^ HD- and AML- MSCs cells (E:T ratio1:1) (HD-MSC n=8, AML-MSC n=7). **(E)** CD33.CAR and Tandem CD33/CD146.CAR CIK cells cytokines production (IFN-γ and IL-2) after co-culture with HD-MSCs or KG-1 cells and tripartite co-culture (E:T ratio 1:3) (IFN-γ n=7, IL-2 n=6). **(F)** Proliferation of CD33.CAR and TandemCD33/CD146.CAR CIK cells after co-culture with HD-MSCs or KG-1 cells and tripartite co-culture with (E:T ratio 1:1) (n=8). **(G)** Proliferation of CD146.CAR-, CD33.CAR- and TandemCD33/CD146.CAR CIK cells co-cultured with CD146^+^ stromal cell lines (HS-5 and HS-27A) or KG-1 cells and tripartite co-culture (E:T ratio 1:1) (n=3). (*P<0.05, **P<0.01, ***P<0.001, Two-way ANOVA analysis with Benjamini and Hochberg’s multiple comparisons test). **(H)** Long-term proliferation of CD33.CAR and Tandem CD33/CD146.CAR CIK cells co-cultured with KG-1 cells, in the presence or not of HD-MSCs layer. After 7 days of culture, cells were collected and quantitative proliferation of CAR CIK cells was determined by flow cytometry (Wilcoxon t-test, n=8). NS, not significant.

To better reproduce the human bone marrow niche, TIME cells were replaced with primary BM-derived, healthy (HD-) and leukemic (AML-) MSCs. All target cells were characterized by elevated and comparable expression levels of CD146, evaluated as percentage of positivity and MFI (**Figure 3A**). At first, we assessed the CD146.CAR CIK cells’ ability to recognize and be activated against MSCs but, despite a limited short-term cytotoxic activity (**Figure 3B**), the long-term effector functions, such as cytokines production and proliferation, resulted to be impaired (**Figures 3C, D**). Furthermore, we analyzed if also CD33.CAR and Tandem CD33/CD146.CAR CIK cells were inhibited by MSCs, both in co-culture and in tripartite co-culture with KG-1 cells. As observed with CD146.CAR CIK cells, even Tandem CD33/CD146.CAR CIK cells displayed absence of cytokines release (**Figure 3E**) and proliferation (**Figure 3F**) when triggered with MSCs. Moreover, we highlighted a potent suppression even of CD33.CAR CIK cells’ later-functions when in presence of MSCs and KG-1 cells, as compared to the sole co-culture with KG-1 cells (**Figures 3E, F**), demonstrating that also well-known and effective CAR CIK cells were impacted by the immunomodulatory action of MSCs.

To further strengthen this hypothesis, we evaluated the proliferation of Tandem CD33/CD146.CAR and CD33.CAR CIK cells in tripartite co-culture with KG-1 and CD146^+^ stromal cell lines (HS-5 and HS-27A). As expected, the CAR CIK cells proliferation after engagement with KG-1 was significantly reduced in the presence of HS-5 and HS-27A cells (**Figure 3G**), confirming their suppressive action.

The more remarkable abrogation of CAR CIK cells in the latest effector functions suggested us that the immunomodulation was a long-term process. Indeed, we examined the fold increase of CD33.CAR and Tandem CD33/CD146.CAR CIK cells proliferation after 7 days of stimulation with KG-1 cells. The addition of MSCs layer induced fold increase reduction for both CARs, in accordance with our previous results, and this further confirmed the immunomodulatory action of MSCs (**Figure 3H**).

### Live-cell imaging confirms immunosuppression of CAR CIK cells by stromal cells

To better investigate the immunosuppressive effect of stromal cells on CAR CIK cells, we performed a time-course analysis of apoptosis mediated by CD146.CAR CIK cells against the different target cells (TIME, HD-MSC and HS-27A cells), measuring the levels of the apoptosis marker caspase 3 by live-cell imaging with Operetta CLS high-content analysis system.

**Figure 4:**
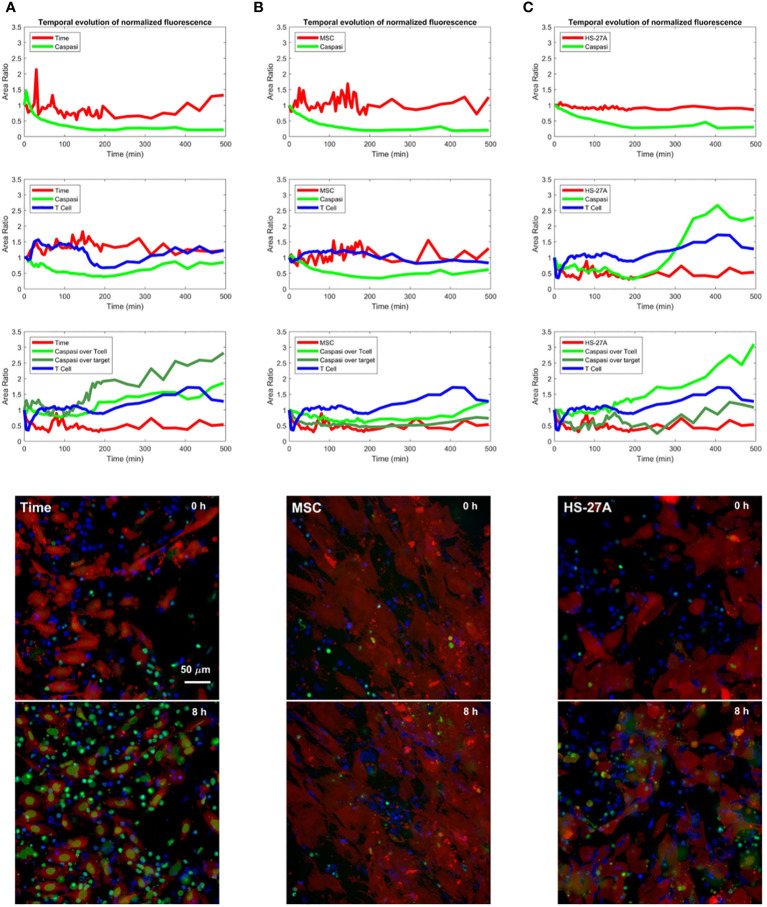
Time-course analysis of apoptosis mediated by CD146.CAR CIK cells. **(A-C)** TIME **(A)**, MSCs **(B)** and HS-27A **(C)** were tested in a time course short-term cytotoxic assay (E:T ratio of 5:1) using Operetta CLS. A custom-made MatLab based software provide the image analyses to extract the populated area of the different dyes. Images show co-culture of different targets (TIME, MSCs and HS-27A) with CD146.CAR CIK cells. First row represents frame took at time 0, while second row reports frame took at the 8-hours end point.

Preliminary results showed caspase 3 increase in the cytoplasm of TIME cells when challenged with CD146.CAR CIK cells as compared to NT cells (**Figure 4A**). CD146.CAR CIK cells did not induce a caspase 3 increase on HD-MSC (**Figure 4B**) and HS-27A cells. Notably, caspase 3 activation was detected in CD146.CAR CIK cell cytoplasm when challenged with HS-27A (**Figure 4C**).

These differences in the apoptosis of TIME and stromal cells mediated by CD146.CAR CIK cells, further confirm the previously observed results, corroborating the immunosuppressive role of the stromal compartment on CAR CIK cell functions.

## Discussion

The prognosis of patients with relapsed or refractory AML is still poor, despite the advent of many novel targeted therapies in the last decade. The development of CAR T cells and other immunotherapeutic strategies are becoming valid alternatives in AML treatment. However, in contrast to the success obtained with CD19 CAR T cells for B-cell malignancies, the progress in AML has been hindered by several challenges, such as the identification of tumor-specific antigens and disease heterogeneity (34). Moreover, the protection of AML cells from conventional chemotherapy and immunotherapy is mainly mediated by the diverse functional properties of the BM niche. AML TME has been shown to exert an immunosuppressive activity, through cellular interactions, soluble environmental factors and structural components (35). T cell effector functions are inhibited by suppressive immune cells, including myeloid derived suppressor cells and Tregs (36, 37). Immune checkpoints, anti-inflammatory cytokines, chemokines and metabolic changes orchestrate together in causing T cell dysfunctions and exhaustion (38–40). Furthermore, alterations of several niche stromal components, including MSCs, osteoblasts, endothelial cells, adipocytes, peripheral neurons and associated Schwann cells, has been linked to malignancy progression and treatment resistance (7).

More recently, these findings were found to be applicable to CAR T cell therapies, whereby the immunosuppressive activity of TME was identified as a possible mechanism implicated in CAR T cell therapy failure (11, 41, 42). In solid tumors, cancer-associated fibroblasts (CAFs) have been shown to play a crucial role in the establishment of an immunosuppressive TME and in limiting T cell infiltration in the tumor mass, through the deposition of extracellular matrix (ECM) proteins. CAFs express high levels of fibroblast activation protein (FAP), and FAP-specific CAR T cells engineered to secrete ECM-degrading enzymes have been associated with improved CAR T cell function through inhibition of tumor stromagenesis, decrease of tumor vascular density and disrupting spatial orientation of tumor cells (24, 43).

Those results give the potential for future immunotherapeutic approaches that will include anti-stroma CAR T cells combined with either anti-tumor CAR T cells or checkpoint blockade. Stromal targeting with CAR T cells has been proposed for the treatment of multiple myeloma (MM) (11) according to previous evidence of immunosuppressive mechanisms on immune T cells (44–47). In particular, a dual targeting of both malignant plasma cells and CAFs showed to be effective in overcoming CAF-induced CAR-T cell inhibition induced by the MM TME (25).

In the current study we explored the feasibility of the simultaneously targeting of AML blasts and the niche stromal compartment through a bispecific Tandem CAR. As a proof-of-concept model, we selected CD146 as MSC target (6, 26–28),. CD33 was chosen as AML antigen as one of the most validated targets (31, 33). As effector CAR cells we used CIK cells. This is an alternative option over conventional T cells, known to be associated with low GVHD and minimal alloreactivity even in an allogeneic setting, as extensively demonstrated by our and other groups (48, 49). Results demonstrated that CIK cells, engineered with CD146.CAR efficiently displayed effector functions when engaged with CD146^+^ target cells. When incorporated in a CD33/CD146 Tandem molecule, Tandem CAR CIK cells were able to recognize both molecules expressed on two different target cells. Thus, we offer a proof of concept regarding the feasibility of a dual targeting model against two different cell populations, which are both present in the leukemic niche.

To better resemble the human BM niche, CD146.CAR CIK cells were tested against CD146^+^ stromal cell lines (HS-5 and HS-27A) as well as against HD and AML derived MSCs. A decrease in killing activity, a strong inhibition of cytokine production, and of proliferation of CAR CIK cells were observed *in vitro*, thus suggesting a potential involvement of the stromal compartment in the immunomodulation triggered against CAR T cells.

*In vivo* studies are needed to better elucidate the underlying mechanisms, but our findings are not entirely unexpected considering the large bulk of data on the immunomodulating activity of BM TME produced over the last decade (50). MSCs were shown to exert several immunomodulatory functions, both on innate and adaptive immunity, through cell-to-cell contact (51) and paracrine activities with immune cells such as T cells, B cells, natural killer cells, macrophages, monocytes, dendritic cells (DCs) and neutrophils (50).

The main role of MSCs immunomodulation relies on the dampening of inflammation. Indeed, the interaction with DCs can cause a shift from pro-inflammatory Th1 cells to anti-inflammatory Th2 cells, leading to a change in the cytokines profile toward an anti-inflammatory milieu (52). Moreover, MSCs were found to induce Treg from conventional T cells, rather than from expansion of pre-existing natural Tregs, thus suggesting a direct role in mediating Tregs formation (52). Among MSCs-related paracrine mechanisms, the constitutive secretion of indoleamine 2,3-dioxygenase (IDO) has been related to inhibition of allogeneic T cell responses and at the same time to the stimulation of IL-4 secretion in Th2 cells and to a decrease of the IFNγ production by Th1 cells (14, 53). In addition, the direct inhibition of alloreactive CD4^+^ and CD8^+^ T cell proliferation by MSCs seems to be partially mediated by MSC-derived galectin-1 (53). Another paracrine molecule secreted by MSCs is PD-L1, known suppressor of T-cell activation and inducer of T-cell hypo-responsiveness and apoptosis (15).

The role of MSCs immunomodulation in CAR T cell therapies has not been fully clarified yet. A recent publication addressed this issue in the context of CD19 CAR T cell therapy, showing that MSCs inhibit T cell responses without compromising CD19-specific CAR T cell activity, highlighting the possibility that the potent effector functions of CAR T cells could overcome the immunomodulation mediated by MSCs (54). It was recently reported that MSCs suppress the activity of CD19 CAR T against lymphoma cells through the secretion of stannoicalcin-1 (55).

In this work we observed, using a co-culture system, an *in vitro* CAR CIK cell inhibition mediated by stromal cells, particularly in later effector functions, such as cytokines release and proliferation. Therefore, considering these first *in vitro* observations, further investigation is warranted in relevant *in vivo* preclinical models to better clarify the underpinning biological mechanisms of the AML blast-niche crosstalk and the related implications in CAR T cell therapy.

Specifically, the combination of BM niche organoids with the proposed bispecific CAR technology could provide the ideal platform to further evaluate the specificity of this system against human CD146^+^ MSC and CD33^+^ AML cells within a humanized ossicle model (27).

Moreover, future studies are needed to dissect the key factors specifically involved in modulating anti-leukemic CAR T cell proliferation and to test different compounds identified as efficient blockers of these molecules. Considering our results on the immunosuppressive role exerted by MSCs on CAR CIK cells, combinatorial strategies will be considered to interfere in the crosstalk between leukemic cells and the niche. As an example, the development of fourth generation armored CARs (56) may allow to overcome the MSCs’ immunosuppression through secretion of inhibitory drugs (such as anti-IDO inhibitors) which mediate the modulation of the paracrine molecule profiles released by the stromal compartment.

In conclusion, the stromal component of the niche could represent an obstacle to CAR efficacy. Therefore, the development of an approach in which CAR T cells are able to eliminate MSCs or a combinatorial strategy with specific compounds capable to decrease the MSCs immunopotency would likely be beneficial.

## Data availability statement

The original contributions presented in the study are included in the article/supplementary material, further inquiries can be directed to the corresponding author/s.

## Ethics statement

The studies involving human participants were reviewed and approved by the Institutional Review Board of the Ethical Committee of San Gerardo Hospital. Written informed consent to participate in this study was provided by the patients or their guardians.

## Author contributions

GA and CA: contributed to the conception and design of the study, performed the experiments and analysis, wrote the manuscript. AP: contributed to the conception of the study, supervised the experiments and revised the manuscript. DS: contributed to data curation, validation and formal analysis. FM: contributed to data curation, validation and formal analysis. FD: supervised the study and revised the manuscript. AB: supervised the study and revised the manuscript. ST: contributed to the conception and design of the study, performed data curation, validation, methodology, formal analysis and wrote and revised the manuscript. MS: contributed to the conception and design of the study, supervised data curation, wrote and revised the manuscript.
